# Supplementary material for: Multipurpose Prevention Technologies: Oral, Parenteral, and Vaginal Dosage Forms for Prevention of HIV/STIs and Unplanned Pregnancy
Source: Polymers (Basel). 2021 Jul 26;13(15):2450. doi: 10.3390/polym13152450 (PMC8347890; doi:10.3390/polym13152450)
Supplement: Supplementary file 1 [file polymers-13-02450-s001.zip › polymers-1299601-supplementary.pdf]

Supplementary Materials.

Table S1. MPTs in preclinical development.

Product Name/Developer	Indication	Delivery Platform	Development Stage	Active Pharmaceutical Ingredients	Duration of Action	Reference
International Partnership for Microbicides	HIV, HSV-2, Pregnancy	Vaginal tablet	-	Dapivirine, levonorgestrel, acyclovir	8 hours	[1]
Population Council	HIV, HSV-2, HPV, Pregnancy	Intravaginal ring	Preclinical – Early	Levonorgestrel, carrageenan, MIV-150, zinc acetate	28 days	[2,3]
	HIV, HSV-2, HPV	Vaginal insert	Preclinical - Early	Griffithsin carrageenan	8 hours	[4]
Population Council/Oak Crest Institute	HIV, Pregnancy	Intravaginal ring (POD)	Preclinical - Early	Q-Griffithsin, etonogestrel, ethinyl estradiol	90 days	[5]
Oak Crest Institute of Science	HIV, HSV-2	Intravaginal ring (POD)	Preclinical - Advanced	Acyclovir, tenofovir	28 days	[6]
CONRAD Program	HIV, Pregnancy	Copper Intrauterine system	Preclinical - Advanced	Elvitegravir	1 year	-
Research Triangle Institute (RTI)	HIV, Pregnancy	Implant	Preclinical - Early	Tenofovir, levonorgestrel, etonogestrel	7 months	[7,8]
Merck	HIV, pregnancy	Implant	-	Islatravir, etonogestrel	≥ 1 year	[9]
PATH/Queen's University at Belfast	HIV, Pregnancy	Microarray Patch	Preclinical - Early	Cabotegravir, progestin	7 days	[10]
Oak Crest Institute of Science	HIV, HSV-2, Pregnancy	Intravaginal Ring (POD)	Preclinical - Advanced	Tenofovir alafenamide hemifumarate, acyclovir, ethinyl estradiol, etonogestrel	35 days	[11]
SRI International	HIV, HSV-2	Vaginal gel	Preclinical – Early	Tenofovir, acyclovir	24 hours	[12]

References

1. McConville, C.; Major, I.; Devlin, B.; Brimer, A. Development of a multi-layered vaginal tablet containing dapivirine, levonorgestrel and acyclovir for use as a multipurpose prevention technology. *Eur. J. Pharm. Biopharm.* **2016**, *104*, 171–179, doi:10.1016/j.ejpb.2016.05.003.
2. Derby, N.; Aravantinou, M.; Kenney, J.; Ugaonkar, S.R.; Wesenberg, A.; Wilk, J.; Kizima, L.; Rodriguez, A.; Zhang, S.; Mizenina, O.; et al. An intravaginal ring that releases three antiviral agents and a contraceptive blocks SHIV-RT infection, reduces HSV-2 shedding, and suppresses hormonal cycling in rhesus macaques. *Drug Deliv. Transl. Res.* **2017**, *7*, 840–858, doi:10.1007/s13346-017-0389-0.
3. Ugaonkar, S.R.; Wesenberg, A.; Wilk, J.; Seidor, S.; Mizenina, O.; Kizima, L.; Rodriguez, A.; Zhang, S.; Levendosky, K.; Kenney, J.; et al. A novel intravaginal ring to prevent HIV-1, HSV-2, HPV, and unintended pregnancy. *J. Control. Release* **2015**, *213*, 57–68, doi:10.1016/j.jconrel.2015.06.018.
4. Derby, N.; Lal, M.; Aravantinou, M.; Kizima, L.; Barnable, P.; Rodriguez, A.; Lai, M.; Wesenberg, A.; Ugaonkar, S.; Levendosky, K.; et al. Griffithsin carrageenan fast dissolving inserts prevent SHIV HSV-2 and HPV infections in vivo. *Nat. Commun.* **2018**, *9*, 1–9, doi:10.1038/s41467-018-06349-0.
5. An Etonogestrel/Ethinyl Estradiol/QGriffithsin (ETG/EE/QGRFT) IVR to Prevent Pregnancy and HIV. Available online: https://reporter.nih.gov/search/d-lshHFKJUaKMHAXEj_7PA/project-details/10138979 (accessed on 31 May 2021).
6. Moss, J.; Malone, A.M.; Smith, T.J.; Kennedy, S.; Kopin, E.; Nguyen, C.; Gilman, J.; Butkyavichene, I.; Vincent, K.; Motamedi, M.; et al. Simultaneous Delivery of Tenofovir and Acyclovir via an Intravaginal Ring. *Antimicrob. Agents Chemother.* **2011**, *56*, 875–882, doi:10.1128/aac.05662-11.
7. Li, L.; Krovi, S.; Norton, C.; Luecke, E.; Demkovich, Z.; Johnson, P.; Areson, C.; Jimenez, G.; van der Straten, A.; Johnson, L. Biodegradable Implant for Sustained Delivery of Antiretroviral (ARV) and Hormonal Contraceptive. Presented at Conference on Retroviruses and Opportunistic Infections (CROI), Boston, MA, USA, March 2020.
8. Johnson, L.; Li, A.; Krovi, S.; Luecke, E.; Demokovich, Z.; Johnson, P.; Shapely-Quinn, M.; van der Straten, A. Biodegradable Implants for HIV Prevention and Contraception. Presented at Long-Acting/Extended Release Antiretrovial Resource Program (LEAP), Boston, MA, USA, March 2020.

9. Grobler, J. Current status of the Merck LA/ER Pipline: Update on Islatravir (ISL, MK-8591). Presented at Long-acting/Extended Release Antiretroviral Resource Program (LEAP), Boston, MA, USA, March 2020.
10. Rein-Weston, A.; Tekko, I.; Vora, L.; Jarrahian, C.; Spreen, B.; Scott, T.; Donnelly, R.; Zehrung, D. LB8. Microarray Patch Delivery of Long-Acting HIV PrEP and Contraception. *Open Forum Infect. Dis.* **2019**, *6*, S996, doi:10.1093/ofid/ofz415.2491.
11. Smith, J.M.; Moss, J.; Srinivasan, P.; Butkyavichene, I.; Gunawardana, M.; Fanter, R.; Miller, C.S.; Sanchez, D.; Yang, F.; Ellis, S.; et al. Novel multipurpose pod-intravaginal ring for the prevention of HIV, HSV, and unintended pregnancy: Pharmacokinetic evaluation in a macaque model. *PLOS ONE* **2017**, *12*, e0185946, doi:10.1371/journal.pone.0185946.
12. Fields, S.A.; Bhatia, G.; Fong, J.M.; Liu, M.; Shankar, G.N. SR-2P Vaginal Microbicide Gel Provides Protection against Herpes Simplex Virus 2 When Administered as a Combined Prophylactic and Postexposure Therapeutic. *Antimicrob. Agents Chemother.* **2015**, *59*, 5697–5704, doi:10.1128/aac.00690-15.
